# Workplace-based assessments for postgraduate training in neonatal intensive care unit – a qualitative study of the perceptions of trainers and trainees

**DOI:** 10.1186/s12909-026-09228-1

**Published:** 2026-04-17

**Authors:** Ayevbekpen Grace Okoye, Camille A M Huser

**Affiliations:** 1 Neonatal Intensive Care Unit, King’s College Hospital NHS Foundation Trust, London, UK; 2 University of Glasgow, Undergraduate Medical School, Room 443, Glasgow, UK

**Keywords:** Workplace-based assessments, Neonatal intensive care unit, Trainees, Trainers, Learning, Feasibility, Acceptability, Paediatric, Postgraduate education, Outcome-based education

## Abstract

**Background:**

As paediatric postgraduate education in the UK is pivoting its structure to further align with an outcome-based training model, the perception of the feasibility, acceptability and learning impact of workplace-based assessments (WBAs) in this new curriculum structure needs to be understood. Neonatal intensive care training is an essential component of general paediatric training, and provides a markedly different learning environment than other paediatric specialities. This study aimed to uncover the perceptions of trainees and trainers on WBAs in this particular context.

**Methods:**

An exploratory qualitative study was conducted, using semi-structured interviews of six trainees, and a semi-structured focus group with four trainers, in one UK hospital. Inductive thematic analysis was undertaken to tease out the main themes.

**Results:**

Five main themes were identified: impact on learning, self-directed learning, approach to WBAs, acceptable learning and assessment tool in the neonatal intensive care unit (NICU), and feasibility of WBAs.

**Conclusions:**

The findings of this study can inform the implementation of WBAs within the current structural curriculum in the paediatric postgraduate education in the UK, to ensure the WBAs remain meaningful and impactful, acceptable and feasible.

**Supplementary Information:**

The online version contains supplementary material available at 10.1186/s12909-026-09228-1.

## Background

The postgraduate paediatric curriculum in the UK is set and overseen by the Royal college of paediatrics and child health (RCPCH). The curriculum is based on trainees completing learning outcomes at each stage of training in line with competency-based medical education (CBME). CBME can be defined as gaining skills, knowledge, and attitudes with emphasis on achieving a set of competencies within a defined curriculum [1, 2]. It is at times used interchangeably with outcome-based education (OBE) as there are similarities such as ‘outcomes’ and ‘competencies’ which the trainees have to achieve. In CBME, assessments are linked to achievement of competencies [3]. CBME is an approach to education where direct observation of clinical performance in the workplace is crucial [4]. The upcoming RCPCH “Progress +” [5] provides a restructuring of the training programme to align with an CBME framework in response to the shape of training towards better patient care [6].

A key component of CBME is the use of workplace-based assessments (WBAs) [7, 8] and these should be an important focus during this transition. WBAs are assessment tools used in the clinical environment with the aim not only of assessing performance, but with a focus on stimulating learning and engagement with feedback [9]. WBAs are therefore clearly assessments for learning, rather than assessments of learning. Typical examples of WBAs include the mini-clinical evaluation exercise (mini-CEX), direct observation of procedural skills (DOPS), case-based discussions (CbDs) and multi-source feedback (MSF) [9]. Unlike assessments such as objective structured clinical examinations (OSCEs) which measure competence and align with the ‘shows how’ level of Miller’s landmark paper [10, 11], WBAs measure performance and align with the top ‘does’ level [12], and the ‘is’ level linked to professional identity [13].

In the United Kingdom, paediatric specialty training follows an eight-year structured programme (Specialty Training (ST) year one to eight) governed by the RCPCH. WBAs are an integral part of this process and include Supervised Learning Events (SLEs), which are formative, supervised encounters designed to stimulate feedback and reflection; the mini-CEX, which assesses clinical encounters; DOPS, which evaluates technical and procedural competence; CbD, which explores clinical reasoning and decision-making, as well as MSF, which collates perspectives from colleagues on professional behaviours and teamwork [9, 14]. All WBAs are documented within the RCPCH ePortfolio, an online platform used to record feedback, reflection and evidence of progression for the Annual Review of Competence Progression (ARCP) [5, 6]. The ePortfolio therefore functions as both an educational and administrative record throughout training.

All WBAs are characterised by trainers’ direct observation, judgement on trainees’ performance against a standard, assessor feedback, identification of potential strengths and learning needs [9, 13]. It is important to note that WBAs have both summative and formative function in trainees’ progress and development. Summative assessment is ‘high stakes’ and makes a judgement of trainee progression to the next stage whilst formative assessment is ‘low stakes’ and intended to enable learning [13]. The use of formal feedback to direct trainees’ learning is an important component of assessment for learning and a goal of WBAs. Assessment for learning is a theory that blends constructs from ‘learner autonomy’, self-assessment, active learning, learner goals and objectives, reflective practice, and feedback [15]. Various authors demonstrated the importance of assessment for learning in supporting students’ progress and achievements making them self-directed learners [15–18].

The ‘consensus criteria’ set at Ottawa Conference in 2010 stipulate set standards and recommendations to judge the quality of assessments [14]. This study examines three of the Ottawa criteria for WBAs in the neonatal intensive care unit (NICU) clinical setting: feasibility, acceptability and catalytic effect (how the assessment drives learning).

### Significance of this study and justification

In an OBE model, there should be emphasis on support, supervision, and assessments to ensure trainee progression is efficient. Rao [19] postulates that ‘the most important aspect of an outcome is that it should be observable and measurable’. WBAs in the clinical environment have been implemented and used for this purpose.

Despite the postgraduate paediatric training pivoting to an OBE model in the Progress plus curriculum [6], where WBAs will play a critical role in learning and progression decisions, there is limited empirical data on the quality of WBAs within the NICU setting. NICU training offers fundamental knowledge and skills needed at the end of paediatric training and consolidates the competences of future paediatricians. Furthermore, the views of relevant users such as trainees are important in appraising WBAs [20]. It is therefore timely and important to examine how WBAs are perceived in this context by both trainees and trainers, and results from this study can be used to ensure that WBAs remain relevant and effective in the new training model.

## Methods

### Study aim and design

The aim of this study was to explore trainees and trainers’ perceptions of WBAs in the NICU and gain understanding of the feasibility, acceptability and learning impact of WBAs in the busy NICU clinical setting within an outcome-based postgraduate training programme in England. Specifically, it aimed to answer three research questions within this context:

1. What factors related to WBAs impact the learning experience of trainees in a NICU setting?
2. How do the factors identified in question 1 impact the learning experience of trainees in a NICU setting?
3. What are trainees’ and trainers’ perceptions of the feasibility and acceptability of WBAs in a NICU setting?

Due to the paucity of existing studies on WBAs in this specific context, an exploratory qualitative study design was adopted using semi-structured individual interviews with trainees and a focus group discussion with trainers.

### Ethics approval and consent to participate

Ethical approval was granted from the University of Glasgow (Project 200220150) and the study was designed and conducted in accordance with the Declaration of Helsinki. Clinical trial number: not applicable. Informed consent was obtained from all participants in the study including the pilot participant.

### Participants

Study participants were trainees and trainers working in a single UK neonatal intensive care unit. Convenience sampling was used, with participation voluntary [21, 22]. Seventeen trainees were eligible to participate; six consented, representing a response rate of 35%. Fourteen trainers were eligible; four participated, representing a response rate of 28%. Recruitment was conducted via email sent by an administrator, with three reminder emails issued. No participants withdrew after consenting.

Of the six trainee participants, four were female and two were male, spanning training levels from ST2 to ST8 (female: ST2, ST3, ST6, ST7; male: ST3, ST8). Among the four trainer participants, three were female and one male; all were consultant neonatologists with between five and fifteen years’ experience as consultants and educational supervisors.

### Data collection

Semi-structured interviews were used to explore the perceptions and opinions of trainee participants on the phenomenon [21], conducted online using Zoom, and recorded [23, 24]. No existing interview guide was identified as suitable for the study context. Following a literature review, interview questions were developed to address the research questions and mapped to the Ottawa Conference criteria of feasibility, acceptability, and learning impact. Separate semi-structured interview guides for trainees and trainers are provided as supplementary material (Supplementary Files 1 and 2).

A pilot individual interview was conducted, and the interview guide minimally modified. Data from the pilot interview were not included in the final analysis.

To allow for a level of interaction between participating trainers [25], a focus group approach was used to gather their perceptions and opinions, which was conducted online using Zoom, and recorded. A focus group guide was prepared following the same review of literature and alignment to research questions and Ottawa criteria.

Six interviews and one focus group with four trainer participants were conducted.

All recordings were deleted once transcripts were finalised, member checked to ensure rigour [26–29] by allowing participants to review the transcripts of their interviews for completeness and accuracy, and anonymised. All interviews and the focus group were conducted remotely via Zoom in private environments, with no non-participants present. Interviews lasted approximately 30–45 min, and the focus group approximately 60 min. Sessions were audio-visually recorded with participant consent and professionally transcribed verbatim. Field notes were made following each interview and focus group to support reflexivity.

### Data analysis

Inductive thematic analysis was used to extract themes from the interviews and focus group, following Braun and Clarke’s six steps method [29]. Following familiarisation with the data, initial inductive coding was undertaken (Braun and Clarke phase 2), with codes generated directly from the data and iteratively refined as analysis progressed. Codes were grouped into sub-themes, which were further grouped into key themes. In an iterative process, sub-themes were reviewed and revised to accurately reflect the data. Rigour was ensured through independent code and theme generation from the two researchers until consensus was achieved. Consensus was reached when the themes were deemed to be distinct from each other yet represent all the data in a meaningful and useful manner by both researchers. Triangulation was used to identify themes in both trainees and trainers’ data. Trainee interview transcripts were coded first, with codes generated directly from the data and refined iteratively as subsequent transcripts were analysed. Trainer focus group data were then coded separately using the same inductive approach. Once both datasets had been analysed independently, themes were compared across trainee and trainer data to identify areas of convergence and divergence. NVivo 12 software was used to manage data and maintain an audit trail, and themes were reviewed and refined through iterative discussion between authors until consensus was achieved. Formal data saturation was not assessed; instead, sufficiency of the dataset was determined by the exploratory study design, narrow aim of the study and homogeneity of the participant population, as well as recurrence of patterns across interviews and convergence of themes between trainee and trainer data [22].

### Researcher positionality (Reflexivity)

The interviews and focus group were conducted by AGO, a consultant neonatologist with an MRes in Health Professions Education. Both authors (AGO and CH) are female and experienced medical educators; CH is a Professor of Medical Education at the University of Glasgow. At the time of the study, AGO worked within the participating neonatal intensive care unit but did not directly supervise or assess any of the participating trainees, and CH had no clinical role within the unit. No prior personal relationships existed between the researchers and participants, and interviewer characteristics were not disclosed to participants to minimise response bias.

AGO occupied an insider researcher position as a trainer within the study setting [30]. The potential influence of this positionality on data collection and analysis was explicitly acknowledged and addressed through reflexivity throughout the research process [31–35]. This included conscious reflection on how prior experiences, assumptions, and beliefs might shape the formulation of the research questions, development of the interview guides, conduct of interviews, and interpretation of data. Reflexivity was supported through the use of field notes following each interview and focus group, and through ongoing analytic discussion between authors. To further enhance rigour and minimise bias, CH independently reviewed the analysis and undertook separate coding of the data, with differences discussed until consensus was achieved.

## Results

Analysis of six trainee interviews and one trainer focus group generated five overarching themes. Themes are presented with illustrative quotations from both trainee and trainer data, with areas of convergence and divergence highlighted where relevant. A total of 97 codes were identified, which were further classified into 12 sub-themes, themselves further grouped into 5 overarching key themes:

- Impact on learning
- Self-directed learning
- Approach to workplace-based assessments
- Acceptable learning and assessment tool in the NICU
- Feasibility of workplace-based assessments in the NICU

A coding tree is provided as Supplementary file 3.

### Impact on learning

Participants viewed WBAs as playing an important role in learning within an outcome-based postgraduate training placement in the NICU. Factors that particularly influenced learning included feedback, the clinical learning environment, supervision, reflection and support from trainers. All trainees reported that feedback following WBAs played a significant role in their learning. They discussed its usefulness, quality and timeliness, with immediate feedback perceived as particularly valuable because it helped identify both areas for improvement and progress in learning. One trainee commented:

*the most learning impact is actually sitting down straight away and giving direct feedback at that moment….I find the assessments useful but I think it has to be in conjunction with direct and immediate discussion with the trainee [TE1]*.

Conversely, generic feedback was not perceived as constructive. One trainee commented:

*Some [supervisors] may just say well done. So then you don’t get much out of it. But if the assessor actually goes into much detail*,* for example*,* okay*,* this is what you did well*,* this is what you can improve in*,* then there is value in learning. [TE5]*

Both trainees and trainers described the NICU as offering numerous opportunities for WBAs because of the large number of clinical procedures performed in this setting. One trainee summarised this view:

*I think realistically there are many opportunities to do different types of workplace-based assessments [TE1]*.

Participants also described WBAs as ‘opportunistic learning’ [TR2] within the NICU environment. However, although the complexity and busyness of the NICU created many opportunities for WBAs, participants perceived that dedicated time outside routine clinical work was often needed to complete them fully. One trainer commented:

*You can always do opportunistic workplace-based assessments as they arise. But if you are going to do it [WBAs] properly*,* I suppose you need to dedicate that time and has to be outside of the clinical service provision that we [trainees] do [TR2]*.

In addition, the presence of supervisors in some NICU areas was perceived to support trainees’ confidence by ensuring that their clinical performance had been directly observed. One trainee reported:

*… that has given me the confidence*,* knowing that actually it’s something that someone has physically seen me do and they [supervisors] know I can do it [TE4]*.

With regards to reflective learning, trainers described how they encouraged trainees to reflect through WBAs and take responsibility for their learning. Trainees also alluded to the role of WBAs in encouraging reflection.

All the trainees described trainers as being supportive and approachable. However, the busyness of NICU and availability of supervisors were perceived as challenges to complete WBAs. One commented:

*… I have been able to approach people [supervisors] for assessments quite easily*,* and that has never been a problem*,* but sometimes again*,* not very often*,* but sometimes I had to send many reminders for it [WBA]*,* and the reminders have not worked. [TE3]*

Trainers echoed their willingness to see trainees progress and to encourage trainees’ learning. One trainer highlighted:

*… you are acting as somebody who should facilitate their [the trainees] progress through their [the trainees] career [TR2]*.

### Self-directed learning

This overarching theme depicts trainees taking ownership of their learning through WBAs within the NICU and how an outcome-based system influences this process. The main factors described were motivation and the trainee’s attitude towards WBAs. The general consensus amongst participating trainers was that for WBAs to be successfully completed, trainees needed to have an active role. There was discussion about trainees who planned WBAs in advance, an attitude that leads to meaningful achievements:

*I think the trainee has to go into a rotation with certain goals in mind*,* because I think that really helps. and that helps by defining goals… So I think planning well in advance………*,* for example*,* I want to learn this procedure*,* and I want to get it assessed so that I can demonstrate like a linear progression is important [TE3]*.

All the trainers recognised that some trainees complete WBAs for learning, preparing and planning to make the most of it, whilst some completed them as a tick-box exercise. One trainer reported:

*I think that it is very variable how useful they [WBAs] are because it would depend on each of the trainees. Some of them take it [WBAs] very seriously*,* and not as a tick box exercise and will choose good cases. [TR3]*

Despite an acknowledgment that there can be a drive to treat WBAs as a tick-box exercise because of demands to complete these assessments on time for the postgraduate exam (ARCP), the trainees who participated in this study took WBAs seriously.

### Approach to workplace-based assessments

The overarching theme described the participants’ approach to completion of WBAs. Both trainers and trainees reported the importance of completion of WBAs for training progression and for the annual appraisal process. The factors which influenced their approach were curriculum and training outcomes, and types of WBAs. All trainees discussed the connections between completion of WBAs and meeting training outcomes. They expressed this requirement was necessary but was not beneficial for learning. One trainee commented:

*and if you were just rushing at the end of your rotation just to try and get to ARCP [annual review of competency progression]*,* I don’t think that makes it [WBAs] beneficial at all. [TE4]*

Trainers echoed this perception. They felt that the requirement to complete a particular number of WBAs to progress to the next level of training overshadowed their learning value. One trainer commented:

*So what I have experienced is that over the course of introducing these SLEs [supervised learning events]*,* we have increased in the number of what [WBAs] we are actually asked to do nowadays. So you add one [WBA]*,* you add one*,* you add one*,* so you actually then become a pure paper-based exercise*,* and there is no kind of any other way you can actually reflect. [TR4]*

DOPS and CbDs were the most popular WBAs according to trainers. DOPS can be undertaken frequently as procedures are undertaken often in the NICU. Similarly, the NICU setting offers ample choice of complex cases to discuss in a CbD. Trainees’ views mirrored that of trainers:

*most of our DOPs are based in NICU. So like*,* airway management*,* putting in UAC/UVCs and things like that which are only in the NICU setting. So I think it tends to be easier to get the practical procedure signed off’. [TE2]*

### Acceptable learning and assessment tool in the NICU

This overarching theme represented participants views and perceptions of WBAs as acceptable learning and assessment tools within the NICU. There were unfavourable views about how learning and competency was captured through completion of the assessment forms, as participants expressed the limitations of WBAs to aid reflection and identify trainees’ level of competence through the ePortfolio.

All participants acknowledged that on the NICU, WBAs are an acceptable learning and assessment tool. However, trainers felt some of the tools were not used properly by trainees, including MSF, as the trainees don’t make use of a broad range of experienced raters to gather comprehensive feedback. Trainers and trainees expressed very similar views on the structure of the ePortfolio, where completion of WBAs is recorded. Participants reported that some of the WBAs forms were cumbersome to complete, the questions on the form discouraged reflection and provided little information about the learning achieved. For these reasons, there was a general agreement that the ePortfolio is not wholly able to capture all learning and evidence competency. One trainer commented:

*in our experience as well that there are so many trainees that actually are struggling trainees. They go through the system because they have managed to fill in the ePortfolio. But in real life they are struggling*,* they are finding it difficult. They are actually not a suitable candidate to be a consultant*,* but the ePortfolio doesn’t pick it up. [TR4]*

### Feasibility of workplace-based assessments in the NICU

This overarching theme describes the perceived feasibility of WBAs within the NICU environment. Participants characterised the NICU as a busy clinical setting caring for complex patients, which often restricted the time available to complete WBAs. However, some features of the NICU environment, such as consultant presence on night shifts, were also highlighted as beneficial to WBAs completion.

It was unanimously agreed by all trainers that completion of WBAs on the NICU was hard to fit into daily clinical activities due to lack of time to observe trainees and complete WBAs electronically. Some trainers suggested it is not possible to do it properly within working hours whilst others felt it shouldn’t be done in one’s own time. Similarly, trainees felt the expectation was to undertake WBAs in their own time.

Trainees shared views about the impact of a lack of time. In particular, lack of time prevented immediate meaningful discussions with the trainer after WBAs, hence missing out on immediate feedback and learning. One trainee remarked:

*If you’ve got the time to go to an office and sit down for a chat*,* that’s brilliant. If you’ve got to try*,* you know you can have the chat on the job*,* and then you can send an assessment on the train home. But that’s less useful*,* that probably negates the usefulness [of the WBA] a little bit. [TE6]*

A constant recurring theme was the busyness of the NICU. Some trainees made comparison to other specialities and classed the NICU as very busy, with high patient acuity and workload which made it difficult to complete WBAs. Although these aspects of the NICU can make completion of WBAs difficult, some trainers and trainees perceived the NICU environment as beneficial. Trainees stated they got the most learning from completion of WBAs during night shifts. Trainers who do night shifts felt there was more focus on learning from WBAs and more time for trainees. One trainer remarked:

*…most of our registrars and SHOs [senior house officers] said that the most they got from their [WBAs] kind of learning was actually at night because there was that time to sit down and not during the day. [TR2]*

## Discussion

This study suggests that several factors influence trainees’ learning experiences with workplace-based assessments in the NICU setting, including feedback, supervision, reflection, support from trainers, and the learning environment.

Feedback has been identified as a significant factor for learning within recent literature on WBAs [36–39]. Jefferies’ qualitative study [38] demonstrated that amongst paediatric trainees, feedback was an essential factor that impacts trainees. Cheston et al.’s large mixed study [37] demonstrated the widespread negative impact of a lack of immediate feedback on trainees’ learning over a long period of time in a range of specialties. Nathoo et al. [39] posit feedback as the most important factor towards trainees’ learning.

The findings in our study on feedback are consistent with current literature findings that immediate and constructive feedback is more beneficial than generic and delayed feedback [36, 38, 40, 41]. It has been demonstrated in UK based paediatric studies that the quality of feedback and timing does impact learning experience [38, 41], although the latter study only reviewed CbDs. Our findings suggested that the immediacy of feedback was influenced by the type of WBA performed, with DOPS easier to complete at the time of the procedure and direct feedback given, consistent with findings from Hunter et al. [42].

The findings in this study indicated that the NICU environment had particularities in supporting or opposing learning via WBAs. Jefferies [38] and Rees et al. [43] both demonstrated the beneficial effects of a supportive environment on trainee learning. The narrative from our study suggests that the NICU environment is generally supportive, providing numerous opportunities for learning. Having a supportive environment with ample learning opportunities aligns closely with assessment for learning [44]. Our study identified the direct presence of senior clinicians in the NICU as contributing to a supportive learning environment, a finding that has received limited attention in the literature on WBAs. It aligns with other studies which indicated that direct observation is beneficial to learning in other contexts [38, 40, 45–47], and with the assessment for learning pedagogy [44].

However, our findings also indicate that the NICU context is perceived to offer little time for WBAs completion. Although there are no similar studies conducted in the NICU in the literature, several UK based studies agree with the impact of lack of time and clinical demands which affect completion of WBAs and delay feedback [37, 38, 41, 42, 48, 49]. Three of the studies had similar participant cohorts to this study [38, 41, 42] and all but one41 reviewed several WBAs. A US-based dermatology quantitative study analysed the opinions of training program directors and identified the negative impact of lack of time on feedback which aligns with findings in this study [40].

Our study suggested trainers’ willingness to facilitate trainees’ learning through reflection and direct supervision of clinical performance which supports a competency-based approach. We found that trainees perceived a benefit and gained confidence from direct observation and supervision which enabled learning and reflection through the discussion of WBAs with trainers. Jefferies’ [38] and Simmons et al.’s [47] recent studies observed similar findings. A supportive learning environment is associated with motivation and a learning attitude towards WBAs according to self-determination theory [50] and adult learning theory. However, trainee motivation and self-directed learning regarding WBAs in postgraduate training merits further research.

Feasibility in this study refers to the extent to which WBAs can be undertaken within the time, workload, and organisational constraints of the NICU clinical environment [14] and includes the impact of availability of time on completion of WBA forms electronically and the time to do the actual assessments. This study highlighted some challenges faced when clinical service provision and education of postgraduate trainees are competing goals. Although our sample size is small, the social context represents a very busy setting with the findings potentially relevant to similar settings. Studies in surgery, anaesthesia and paediatrics described the significant impact of lack of time in relation to completing assessments [38, 41, 45, 51].

Our findings indicated that participants perceived WBAs to be acceptable assessment tools in the NICU within an outcome-based postgraduate education programme, but with caveats. Trainers in particular felt the use of the ePortfolio to record WBAs does not differentiate competent from struggling trainees. This is consistent with findings in current literature that discuss remediating struggling trainees through assessments such as WBAs [36, 48]. WBAs as assessment tools should assess clinical performance and hence gauge trainees’ competency [9] which aligns with principles of CBME [3] ensuring the set competencies defined in a curriculum can be accurately assessed.

Our results suggest that WBAs in the NICU are sometimes viewed as tick-box exercises as there was a perceived need to complete a certain number of WBAs to proceed with training, a finding consistent with current literature [36–38, 48, 49, 52], but which goes against CBME. The importance of assessment for learning should be reinforced within the training programme to allow WBAs to remain acceptable learning tools.

### Limitations

The findings should be interpreted in light of the study’s exploratory design, small sample size, and single-centre context. While the dataset provided sufficient depth to address the study aims, it is possible that less common or divergent perspectives were not captured.

Our study took place in one centre, and the sample size is small, limiting transferability of our findings to other clinical specialties or contexts. Whilst data saturation was not assessed, data sufficiency and analytic adequacy was judged through recurrence of patterns across interviews and convergence between trainee and trainer perspectives, which was considered sufficient for the exploratory aims of the study. This study explored perceptions on all WBAs undertaken in the NICU, which may limit findings related to a specific WBA.

To assess the quality of assessments, we used the Ottawa Conference criteria [14]. However, our study design only allowed us to examine three of the criteria: feasibility, acceptability and catalytic effect (how the assessment drives learning) of WBAs. For a more comprehensive evaluation of the role of WBAs in CBE in the NICU setting, all criteria should be examined.

## Conclusions

Trainees perceived that feedback on WBAs in the NICU is most meaningful to learning when it is immediate and constructive. In addition, the role of supervision, reflection and learning opportunities was perceived as important to the success of WBAs for learning in the NICU outcome-based postgraduate programme. In alignment with adult learning theory, another perceived essential factor in trainees’ learning is their motivation and active involvement, which participants felt could be encouraged and supported by trainers. In our context, completion of WBAs is necessary for progression which is perceived to overshadow their role in learning. The availability of time and use of the ePortfolio system were perceived to influence the completion of WBAs assessments. Overall, WBAs were perceived as acceptable and potentially feasible assessment tools in this context when approached as learning opportunities and supported by timely feedback and supervision.

### Implications for practice

To improve their learning value and ensure WBAs in the NICU continue to be relevant and beneficial to trainees in the new outcome-based curriculum, WBA forms could be designed to facilitate immediate feedback and reflection following a clinical encounter. In particular, WBAs such as DOPs and CbD that enable immediate feedback and provide assessment for learning may be prioritised. This may offer a more efficient approach to supporting assessment of trainees’ competence to meet the demands of effective patient care within the confines of a busy clinical setting. Trainees on the NICU may be encouraged by trainers, college tutors and programme directors to be autonomous in their own learning by setting learning goals before undertaking WBAs during a clinical encounter. Promotion of trainees’ active involvement and self-directed learning may help reinforce the formative value of WBAs as assessments for learning within an outcome-based training programme. As a result of the time constraints that impact electronic completion of WBAs within the NICU, there may be value in improving current technology to promote a seamless educational encounter between trainee and trainer.

### Recommendations for future research

This study focused on feasibility, acceptability and catalytic effect of WBAs. For a more comprehensive evaluation of WBAs’ purpose in the NICU, studies which evaluate other Ottawa criteria [14] such as validity, reproducibility, equivalence and educational effect are required.

This study also focused on trainees and trainers’ perceptions of WBAs in one paediatric sub-specialty, neonatal intensive care in one centre. To demonstrate additional evidence of the usefulness and challenges of undertaking WBAs to inform paediatric postgraduate training policies, further research should be undertaken in other paediatric subspecialties and in other localities. Because this study focussed on all WBAs within the clinical context of the NICU, findings relating to less-well documented WBAs in the literature such as Leader CbD, HAT and MSF were described. Future studies addressing stakeholder’s perceptions of these particular WBAs in postgraduate paediatric education will be valuable, and add to these findings. Within a busy clinical setting, there are resource implications towards trainees’ learning so it is important that future studies explore feasibility of WBAs to seek practical solutions on time allocation for assessments for both trainees and trainers.

## Supplementary Information

Supplementary Material 1.

Supplementary Material 2.

Supplementary Material 3.

## Data Availability

The datasets generated and/or analysed during the current study are not publicly available due the sensitive nature of the interviews/focus group but are available from the corresponding author on reasonable request.

## Abbreviations

ARCP: Annual Review of Competency Progression
CbD: Case-based Discussion
CBME: Competency based Medical Education
DOPS: Direct Observation of Procedural Skills
HAT: Handover Assessment Tool
Mini: CEX Mini-clinical evaluation exercise
MSF: Multisource feedback
NICU: Neonatal Intensive Care Unit
OBE: Outcome-based Education
OSCE: Objective Structured Clinical Examination
RCPCH: Royal College of Paediatrics and Child Health
SLE: Supervised Learning Events
TE: Trainee
TR: Trainer
UAC: Umbilical Arterial Catheter
UVC: Umbilical Vein Catheter
UK: United Kingdom
WBA: Workplace-Based Assessment

## References

[1] Anderson L. Competency-based education: what is the issue and why does it matter? Policy Snapshot. Denver, CO: Education Commission of the States; 2017.

[2] Frank JR, Snell LS, Cate OT, Holmboe ES, Carraccio C, Swing SR, Harris P, Glasgow NJ, Campbell C, Dath D, Harden RM. Competency-based medical education: theory to practice. Med Teach. 2010;32(8):638–45. 20662574 10.3109/0142159X.2010.501190

[3] Holmboe ES, Sherbino J, Long DM, Swing SR, Frank JR. The role of assessment in competency-based medical education. Med Teach. 2010;32(8):676–82. 20662580 10.3109/0142159X.2010.500704

[4] Black P, Wiliam D. Assessment and classroom learning. Assess Educ. 1998;5(1):7–74.

[5] Royal College of Paediatrics and Child Health. RCPCH Progress+ curriculum: paediatric specialty postgraduate training. London: RCPCH; 2023.

[6] Greenaway D. Securing the future of excellent patient care: final report of the Shape of Training Review. London: Shape of Training Review; 2013.

[7] Martin L, Blissett S, Johnston B, Tsang M, Gauthier S, Ahmed Z, Sibbald M. How workplace-based assessments guide learning in postgraduate education: a scoping review. Med Educ. 2023;57(5):394–405. 36286100 10.1111/medu.14960

[8] Prentice S, Benson J, Kirkpatrick E, Schuwirth L. Workplace-based assessments in postgraduate medical education: a hermeneutic review. Med Educ. 2020;54:981–92. 32403200 10.1111/medu.14221

[9] Norcini J, Burch V. Workplace-based assessment as an educational tool: AMEE Guide 31. Med Teach. 2007;29(9–10):855–71. 18158655 10.1080/01421590701775453

[10] Miller GE. The assessment of clinical skills/competence/performance. Acad Med. 1990;65(9):S63–7. 2400509 10.1097/00001888-199009000-00045

[11] Wass V, Van der Vleuten C, Shatzer J, Jones R. Assessment of clinical competence. Lancet. 2001;357(9260):945–9. 11289364 10.1016/S0140-6736(00)04221-5

[12] Norcini JJ, McKinley DW. Assessment methods in medical education. Teach Teach Educ. 2007;23(3):239–50.

[13] Cruess RL, Cruess SR, Steinert Y. Amending Miller’s pyramid to include professional identity formation. Acad Med. 2016;91(2):180–5. 26332429 10.1097/ACM.0000000000000913

[14] Norcini J, Anderson B, Bollela V, Burch V, Costa MJ, Duvivier R, Galbraith R, Hays R, Kent A, Perrott V, Roberts T. Criteria for good assessment: consensus statement and recommendations from the Ottawa 2010 Conference. Med Teach. 2011;33(3):206–14. 21345060 10.3109/0142159X.2011.551559

[15] McSweeney K. Assessment for learning: from theory to practice. Maynooth (IE): Teaching Council (Ireland); 2012.

[16] Nicol DJ, Macfarlane-Dick D. Formative assessment and self-regulated learning: a model and seven principles of good feedback practice. Stud High Educ. 2006;31(2):199–218.

[17] Black P. Assessment for learning: where is it now? Where is it going? In: Bryan C, Clegg K, editors. Improving student learning through assessment. Oxford: Oxford Centre for Staff and Learning Development; 2006;(1)9–20.

[18] Gibbs G, Simpson C. Conditions under which assessment supports students’ learning. Learn Teach High Educ. 2005;(1):3–31.

[19] Rao NJ. Outcome-based education: an outline. High Educ Future. 2020;7(1):5–21.

[20] Massie J, Ali JM. Workplace-based assessment: a review of user perceptions and strategies to address the identified shortcomings. Adv Health Sci Educ. 2016;21:455–73. 10.1007/s10459-015-9614-0 26003590

[21] Cohen L, Manion L, Morrison K. Research methods in education. London: Routledge; 2017.

[22] Vasileiou K, Barnett J, Thorpe S, Young T. Characterising and justifying sample size sufficiency in interview-based studies. BMC Med Res Methodol. 2018;18:148. 30463515 10.1186/s12874-018-0594-7 PMC6249736

[23] Fleming J. Recognizing and resolving the challenges of being an insider researcher in work-integrated learning. Int J Work Integr Learn. 2018;19(3):311–20.

[24] Oliffe JL, Kelly MT, Gonzalez Montaner G, Yu Ko WF. Zoom interviews: benefits and concessions. Int J Qual Methods. 2021;20:16094069211053522.

[25] Denscombe M. The good research guide: for small-scale social research projects. London: McGraw-Hill Education; 2017.

[26] Creswell JW, Creswell JD. Research design: qualitative, quantitative and mixed methods approaches. 5th ed. Thousand Oaks, CA: Sage Publications; 2017.

[27] Denzin NK, Lincoln YS, editors. The SAGE handbook of qualitative research. 4th ed. Thousand Oaks, CA: Sage Publications; 2011.

[28] Whittemore R, Chase SK, Mandle CL. Validity in qualitative research. Qual Health Res. 2001;11(4):522–37. 11521609 10.1177/104973201129119299

[29] Braun V, Clarke V. Using thematic analysis in psychology. Qual Res Psychol. 2006;3(2):77–101.

[30] Fleming J. Recognizing and resolving the challenges of being an insider researcher in work-integrated learning. Int J Work Integr Learn. 2018;19(3):311–20.

[31] Olmos-Vega FM, Stalmeijer RE, Varpio L, Kahlke R. A practical guide to reflexivity in qualitative research: AMEE Guide 149. Med Teach. 2023;45(3):241–51. 10.1080/0142159X.2022.2057287 35389310

[32] Brannick T, Coghlan D. In defense of being native: the case for insider academic research. Organ Res Methods. 2007;10(1):59–74.

[33] Berger R. Now I see it, now I don’t: researcher’s position and reflexivity in qualitative research. Qual Res. 2015;15(2):219–34.

[34] Dwyer SC, Buckle JL. The space between: on being an insider–outsider in qualitative research. Int J Qual Methods. 2009;8(1):54–63.

[35] Hammersley M. On the teacher as researcher. Educ Action Res. 1993;1(3):425–45.

[36] Barrett A, Galvin R, Scherpbier AJ, Teunissen PW, O’Shaughnessy A, Horgan M. Is the learning value of workplace-based assessment being realised? Postgrad Med J. 2017;93(1097):138–42. 27486250 10.1136/postgradmedj-2015-133917

[37] Cheston H, Graham D, Johnson G, Woodland P. Changes in UK medical trainees’ perceptions of workplace-based assessments across 10 years. Postgrad Med J. 2022;98(1158):269–75. 33452154 10.1136/postgradmedj-2020-137907

[38] Jefferies K. Factors that may improve paediatric workplace-based assessments: an exploratory study. Arch Dis Child. 2022;107(10):941–6. 35768176 10.1136/archdischild-2022-323937

[39] Nathoo NA, Sidhu R, Gingerich A. Educational impact drives feasibility of implementing daily assessment in the workplace. Teach Learn Med. 2020;32(4):389–98. 32129088 10.1080/10401334.2020.1729162

[40] Kirby J, Archibeque L, Confer L, Baird D. Workplace formative assessment: faculty members’ beliefs. Clin Teach. 2016;13(1):33–7. 25962905 10.1111/tct.12348

[41] Mehta F, Brown J, Shaw NJ. Do trainees value feedback in case-based discussion assessments? Med Teach. 2013;35(5):e1166–72. 23137264 10.3109/0142159X.2012.731100

[42] Hunter AR, Baird EJ, Reed MR. Procedure-based assessments in trauma and orthopaedic training: the trainees’ perspective. Med Teach. 2015;37(5):444–9. 25186849 10.3109/0142159X.2014.956055

[43] Rees CE, Cleland JA, Dennis A, Kelly N, Mattick K, Monrouxe LV. Supervised learning events in the Foundation Programme: a UK-wide narrative interview study. BMJ Open. 2014;4(10):e005980. 10.1136/bmjopen-2014-005980 PMC4202004 25324323

[44] Schuwirth LW, van der Vleuten CP. General overview of the theories used in assessment: AMEE Guide 57. Med Teach. 2011;33(10):783–97. 21942477 10.3109/0142159X.2011.611022

[45] Castanelli DJ, Weller JM, Molloy E, Bearman M. How trainees come to trust supervisors in workplace-based assessment: a grounded theory study. Acad Med. 2022;97(5):704. 34732657 10.1097/ACM.0000000000004501 PMC9028297

[46] Kipen E, Flynn E, Woodward-Kron R. Self-regulated learning lens on trainee perceptions of the mini-CEX: a qualitative study. BMJ Open. 2019;9(5):e026796. 10.1136/bmjopen-2018-026796 PMC6538085 31129583

[47] Simmons M. How are workplace-based assessments viewed by UK psychiatry trainees? Psychiatr Danub. 2013;25(Suppl 2):182–4. 23995172

[48] Brierley DJ, Farthing PM, Zijlstra-Shaw S. Assessors’ and trainees’ perceptions of workplace-based assessments in histopathology. J Clin Pathol. 2018;71(12):1100–7. 30196274 10.1136/jclinpath-2018-205361

[49] Tailor A, Dubrey S, Das S. Opinions of the ePortfolio and workplace-based assessments. Clin Med. 2014;14(5):510. 10.7861/clinmedicine.14-5-510 PMC4951960 25301912

[50] Ryan RM, Deci EL. Self-determination theory and the facilitation of intrinsic motivation, social development and well-being. Am Psychol. 2000;55(1):68. 11392867 10.1037//0003-066x.55.1.68

[51] Beamish AJ, Johnston MJ, Harries RL, Mohan HM, Fitzgerald JEF, Humm G, Rabie M, Nally DM, Gokani VJ, Ali O, Burke J. Real-world use of workplace-based assessments in surgical training. Int J Surg. 2020;84:212–8. 32898664 10.1016/j.ijsu.2020.07.068

[52] Shalhoub J, Marshall DC, Ippolito K. Perspectives on procedure-based assessments: a thematic analysis of semi-structured interviews with UK surgical trainees. BMJ Open. 2017;7(3):e013417. 10.1136/bmjopen-2016-013417 PMC5372077 28341687

