# Supplementary figures and images for: Workplace-based assessments for postgraduate training in neonatal intensive care unit – a qualitative study of the perceptions of trainers and trainees
Source: BMC Med Educ. 2026 Apr 17;26:798. doi: 10.1186/s12909-026-09228-1 (PMC13214304; doi:10.1186/s12909-026-09228-1)

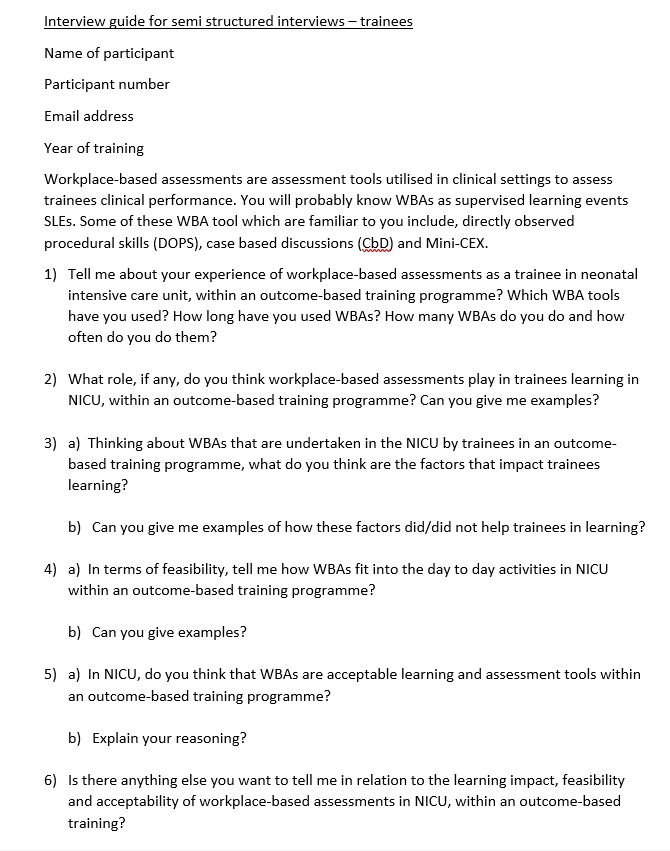

Supplement: Supplementary file 1 — Supplementary Material 1. [file 12909_2026_9228_MOESM1_ESM.docx]

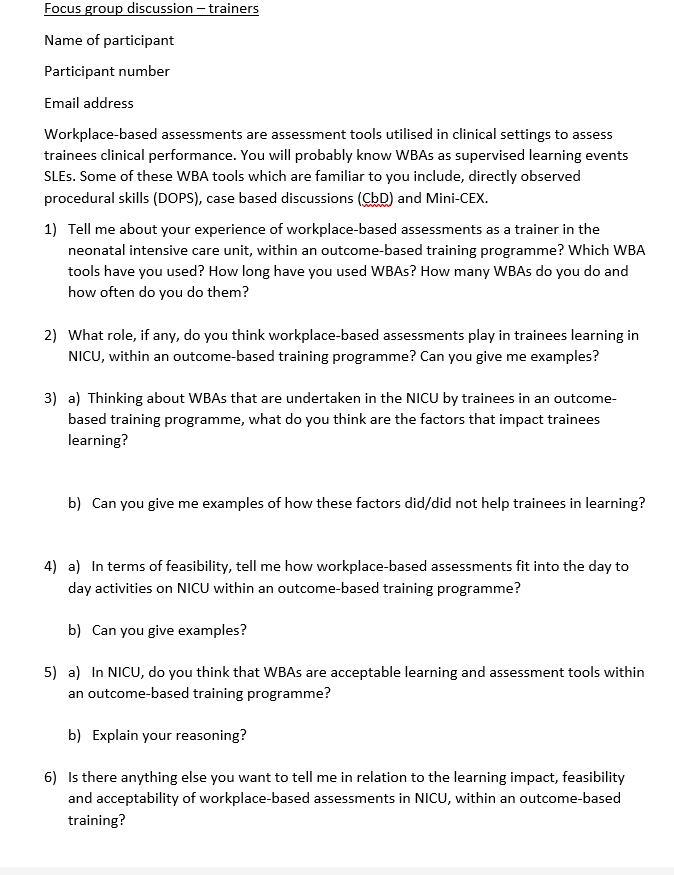

Supplement: Supplementary file 2 — Supplementary Material 2. [file 12909_2026_9228_MOESM2_ESM.docx]
