# Supplementary material for: Workplace-based assessments for postgraduate training in neonatal intensive care unit – a qualitative study of the perceptions of trainers and trainees
Source: BMC Med Educ. 2026 Apr 17;26:798. doi: 10.1186/s12909-026-09228-1 (PMC13214304; doi:10.1186/s12909-026-09228-1)
Supplement: Supplementary file 3 — Supplementary Material 3. [file 12909_2026_9228_MOESM3_ESM.docx]

**Supplementary File 3: Coding tree illustrating inductive thematic analysis**

This coding tree illustrates the inductive development of initial codes into sub-themes and overarching themes following Braun and Clarke’s thematic analysis framework.

**Theme 1: Acceptability of workplace-based assessments**

**Sub-theme: WBAs as an assessment tool**
Codes:
– Objective assessments
– Comparison with other assessment tools

**Sub-theme: Attitudes towards WBAs**
Codes:
– Acceptability
– Familiarity with WBAs
– Format of WBA forms
– Impact of level of training
– Limited information about WBAs
– Perceived purpose of WBAs
– Trainees assessing other trainees
– Trainees’ understanding of WBAs

**Sub-theme: ePortfolio-related factors**
Codes:
– Documentation of learning
– Does not capture all learning
– Does not reflect competence
– Electronic versus face-to-face discussion
– Filling WBA forms
– Perceived purpose of the portfolio
– Portfolio changes over time
– Administrative burden

**Theme 2: Approach to WBAs within training programmes**

**Sub-theme: Mandatory curriculum requirements**
Codes:
– Mandatory WBAs
– Number-driven assessments
– Number of WBAs completed
– Portfolio-driven completion
– Selection of WBAs for ARCP
– Sign-off requirements
– Tick-box exercise

**Sub-theme: Training programme context**
Codes:
– Relevance of outcomes
– Role of the curriculum
– Snapshot of training

**Sub-theme: Types of WBAs**
Codes:
– Case-based discussion (CbD)
– Directly observed procedural skills (DOPS)
– Mini-CEX
– Leader CbD
– Safeguarding CbD
– Multi-source feedback (MSF)
– Trainers’ preferences for WBAs

**Theme 3: Feasibility of WBAs in the NICU**

**Sub-theme: NICU-specific challenges**
Codes:
– Emergency specialty
– Impact of staffing
– Practicality in NICU
– Supervision in a busy NICU
– WBA completion during night shifts
– Workload pressures

**Sub-theme: Time constraints**
Codes:
– Impact of time on WBA completion
– Practicality of time
– Time for preparation
– Fitting WBAs into working time

**Theme 4: Impact of WBAs on learning**

**Sub-theme: Feedback**
Codes:
– Quality of feedback
– Timely feedback
– Useful feedback
– Feedback training
– Retrospective feedback

**Sub-theme: Learning experience**
Codes:
– Learning value of WBAs
– Further reading
– More valuable for learning
– Outcome of discussion with trainers

**Sub-theme: Trainers’ support**
Codes:
– Approachable trainers
– Facilitation of trainee progress
– Supportive trainers
– Team support

**Theme 5: Personal and professional development**

**Sub-theme: Reflection and supervision**
Codes:
– Reflective learning through WBAs
– Opinions on reflection
– Supervision location
– Limited supervision

**Sub-theme: Personal learning**
Codes:
– Engagement and interest in learning
– Motivation
– Planning and organisation
– Role in trainees’ learning
– Self-directed learning
– Self-satisfaction
– Trainee active involvement
